# Reactions

**Published:** 1893-11

**Authors:** 


REACTIONS.—A SELECTION OF ORGANIC CHEMICAL PREPARATIONS IMAORTANT TO PHARMACY IN REGARD TO THEIR BEHAVIOR TO COMMONLY USED REAGENTS, by F. Fluckiger, Ph. D., M. D. Authorized English edition. Translated, revised and enlarged by J. B. Nagelvoort, Analytical Chemist to the Pharm-Chem. Laboratory of Parke, Davis & Co., with portrait and autograph letter of the auuhor. Published by Geo. S. Davis.

The author has given us the chemical reactions for about one hundred and fifty preparations. The work represents the practical results of years of carefully conducted laboratory work by Prof. Fluckiger. Mr. Nagelvoort has done more than simply translate the German text, he has revised and enlarged it, both as the result of his own labor and by the addition of facts obtained from notes furnished by the author.

The book is handsomely gotten up, on the best of paper and has ample marginal space for notes.

